# The Nature of Exposure Drives Transmission of Nipah Viruses from Malaysia and Bangladesh in Ferrets

**DOI:** 10.1371/journal.pntd.0004775

**Published:** 2016-06-24

**Authors:** Bronwyn A. Clayton, Deborah Middleton, Rachel Arkinstall, Leah Frazer, Lin-Fa Wang, Glenn A. Marsh

**Affiliations:** 1 Health and Biosecurity, Australian Animal Health Laboratory, Commonwealth Scientific and Industrial Research Organisation, Geelong, Victoria, Australia; 2 Program in Emerging Infectious Disease, Duke–National University of Singapore Graduate Medical School, Singapore; University of Texas Medical Branch, UNITED STATES

## Abstract

Person-to-person transmission is a key feature of human Nipah virus outbreaks in Bangladesh. In contrast, in an outbreak of Nipah virus in Malaysia, people acquired infections from pigs. It is not known whether this important epidemiological difference is driven primarily by differences between NiV Bangladesh (NiV-BD) and Malaysia (NiV-MY) at a virus level, or by environmental or host factors. In a time course study, ferrets were oronasally exposed to equivalent doses of NiV-BD or NiV-MY. More rapid onset of productive infection and higher levels of virus replication in respiratory tract tissues were seen for NiV-BD compared to NiV-MY, corroborating our previous report of increased oral shedding of NiV-BD in ferrets and suggesting a contributory mechanism for increased NiV-BD transmission between people compared to NiV-MY. However, we recognize that transmission occurs within a social and environmental framework that may have an important and differentiating role in NiV transmission rates. With this in mind, ferret-to-ferret transmission of NiV-BD and NiV-MY was assessed under differing viral exposure conditions. Transmission was not identified for either virus when naïve ferrets were cohoused with experimentally-infected animals. In contrast, all naïve ferrets developed acute infection following assisted and direct exposure to oronasal fluid from animals that were shedding either NiV-BD or NiV-MY. Our findings for ferrets indicate that, although NiV-BD may be shed at higher levels than NiV-MY, transmission risk may be equivalently low under exposure conditions provided by cohabitation alone. In contrast, active transfer of infected bodily fluids consistently results in transmission, regardless of the virus strain. These observations suggest that the risk of NiV transmission is underpinned by social and environmental factors, and will have practical implications for managing transmission risk during outbreaks of human disease.

## Author Summary

Nipah viruses cause outbreaks of severe human disease with high fatality rates. Different patterns of transmission have been associated with recurrent outbreaks caused by Nipah virus (NiV) in Bangladesh, where person-to-person transmission is a major pathway for human infection, compared to an outbreak in Malaysia, where pig-to-human transmission accounted for virtually all human infections. To date, there have been limited comparative studies that address the question of whether this difference may be attributed to differences between geographical isolates of NiV at the genome level, or to other factors in play during human outbreaks of disease. In this report, we employed the ferret, a surrogate human model, to compare features of infection and transmission of NiV isolates from Bangladesh and Malaysia. Our findings indicate that, although differences in levels of shedding are seen between the virus isolates, transmission risk is more likely determined by interactions between infected patients and at-risk individuals; factors that are driven by the social and environmental context within which human disease events occur. Our observations in the ferret have important implications for the implementation of strategies to mitigate transmission risk during outbreaks of NiV infection in people.

## Introduction

Nipah viruses (NiV) isolated from Malaysia (NiV-MY) and Bangladesh (NiV-BD) each cause human disease characterized by febrile encephalitis with high case fatality rates, but they exhibit different epidemiological features [1]. Person-to-person transmission is an important pathway for NiV-BD infection of people [2], while for NiV-MY most patients acquired their infection from domestic pigs [3, 4]. It has been suggested that a greater prevalence and severity of respiratory disease signs in patients infected with NiV-BD may facilitate person-to-person transmission by this strain [2, 5], and exposure to respiratory secretions from patients is reported to be a major risk factor for onward NiV-BD transmission [6, 7]. However, NiV-MY has also been isolated from respiratory secretions [8], and so the factors responsible for different strain attack rates remain poorly understood. In particular, viral transmission occurs within a social and environmental framework that includes co-morbidities such as malnutrition and pre-existing respiratory disease [2], or exposure factors such as levels of patient care and interactions between patients and at-risk individuals [1, 9]; each of these may also play an important and differentiating role in NiV transmission rates.

Oronasal exposure of ferrets to human isolates of NiV results in fulminant infection that recapitulates key features of the human disease [10, 11]. Additionally, patterns of NiV shedding in ferrets [12] are consistent with those observed in people, with virus having been recovered from respiratory secretions and the urine of each species. Furthermore, the ferret is a social animal, making conspecific housing studies both feasible and meaningful in the context of virus transmission mechanisms.

In earlier work conducted in the ferret, we found significantly higher levels of viral RNA in oropharyngeal secretions of animals infected with NiV-BD by comparison with NiV-MY [12], suggesting that the two strains may differ in their replication efficiency at sites of potential relevance to transmission. In a new time-course study, we have specifically assessed the relative tropism and replication efficiency of NiV-MY and NiV-BD for the pharynx and the upper and lower respiratory tract tissues of ferrets exposed to virus via a plausible natural route. We observed more rapid onset of productive infection and also higher levels of virus replication in respiratory tract tissues of ferrets infected with NiV-BD compared to NiV-MY. The observation of strain differences in replication efficiency in tissues of relevance to transmission risk suggests a contributory mechanism for the observed increased incidence of NiV-BD transmission between people compared to NiV-MY.

We then assessed the relative propensity of NiV-BD and NiV-MY for ferret-to-ferret transmission under differing environmental conditions of viral exposure. Firstly, opportunity for natural transmission of infection was provided by cohousing naïve ferrets with animals in which NiV infection had already been established by experimental exposure. Cohousing permitted direct contact of naïve animals with environmental saliva, urine and feces of infected cage mates; interaction through play including wrestling and mouthing; mutual grooming including licking; and sharing of sleeping quarters, food and water containers, with most contact occurring during the incubation period or disease prodrome. Secondly, we provided an alternative transmission opportunity by the addition of direct transfer of oronasal fluids between experimentally-infected and naïve ferrets during known shedding periods, including the time of advanced clinical disease.

Infection of naïve ferrets was not identified for either NiV-BD or NiV-MY after they were cohoused with experimentally-infected animals as described above. However, all naïve ferrets developed acute NiV infection following their assisted and direct exposure to oronasal fluid from animals that were shedding either NiV-BD or NiV-MY.

Our findings for this infection model indicate that, although there may be higher levels of viral shedding with NiV-BD compared to NiV-MY, the attack rate for both viruses may be equivalently low under exposure conditions provided by cohabitation alone. In contrast, the attack rates for each virus are similarly high when there is active transfer of infected bodily fluids. These observations have practical implications for the management of transmission risk with NiV-BD and NiV-MY in the field.

## Methods

### Ethics statement

The studies were approved by the Commonwealth Scientific and Industrial Research Organisation, Australian Animal Health Laboratory Animal Ethics Committee, in accordance with the National Health and Medical Research Council Australian code for the care and use of animals for scientific purposes 8^th^ edition (2013).

### Animals

Outbred ferrets, 12–18 months of age, were randomly assigned to virus infection groups (NiV-BD or NiV-MY) which were then maintained under separated biosafety level-4 (BSL-4) conditions. Animal housing, husbandry, and handling for sample collections were as previously described [10, 13]. Before each study, ferrets were implanted with a LifeChip Bio-Thermo (Destron Fearing, Eagan, MN, USA), and baseline subcutaneous and also rectal temperatures, body weight data, and serum samples were obtained.

After exposure to viral inoculum or potentially infectious secretions, animals were assessed daily for signs consistent with acute NiV infection such as reduced play activity, including by remote scanning of microchip temperature. Ferrets were also anesthetized at selected time-points for collection of clinical samples as outlined below. Animals that reached a predetermined humane endpoint for disease were euthanized as previously described [10, 11], irrespective of when their euthanasia had been scheduled within the study design. Clinical samples and tissues were collected from all animals at euthanasia to assess for viral shedding, viremia, and virus replication.

### Virus inoculums and animal exposures

Virus inoculums used were low-passage human isolates that had been cultured in Vero cells (passage 2 or 3). The NiV-BD isolate originated from the oropharynx of one of 12 people infected during an encephalitis outbreak in Rajbari district, Bangladesh (Nipah Bangladesh/Human/2004/Rajbari, R1; [14, 15]). The NiV-MY isolate was Nipah virus/Malaysia/Human/99, originating from the cerebrospinal fluid of an encephalitic patient; a comparison of the NiV-BD and NiV-MY strains at the nucleotide and amino acid levels is provided elsewhere [15].

Ferrets in the time-course study and donor animals in the transmission studies were anesthetized and exposed to 5,000 TCID_50_ NiV-BD or NiV-MY in 1 mL phosphate-buffered saline (PBS) via the oronasal route as previously described [11, 12]. In each instance, inoculums were back-titrated on Vero cells to confirm the administered dose.

### Study designs

#### Time-course study

Fourteen animals were exposed to each of NiV-BD or NiV-MY, with two ferrets from each group randomly pre-allocated for euthanasia on each of days 1 to 7 post infection (pi).

#### Transmission study–cohabitation

Two animals (donor ferrets) were directly exposed to each of NiV-BD or NiV-MY and individually housed until day (d) 3pi. Pairs of naïve recipient animals were then introduced into each donor cage; recipients remained with the donor until the donor animal was euthanized. Recipient ferrets were monitored for 28 days, including by collection of clinical samples every 48 hrs up to d20 after their introduction to the study, and then euthanized.

#### Transmission study–cohabitation plus direct transfer of secretions

Two donor animals were exposed to each of NiV-BD or NiV-MY and individually housed until d6pi. Pairs of naïve recipient animals were then introduced into each donor cage and remained with the donor until the donor animal was euthanized. In addition, recipients were directly exposed to the oronasal secretions of the respective donor ferret–once at the time of introduction to the donor, and again on the day of euthanasia of the donor. Oral and nasal washes and oral swabs from donor ferrets were uniquely combined in PBS to 3 mL total volume. Mixtures were held on wet ice for the short period of collection, and then administered to recipient animals via the oronasal route as outlined above. Subsamples of each specimen were retained for assessment of the virus dose administered to recipients.

Recipient ferrets were monitored for 30 days, including by collection of clinical samples every 48 hrs up to d14 after their introduction to the study, and then euthanized.

### Sample analysis

Clinical samples comprised whole EDTA-treated blood, nasal washes, oral and rectal swabs and, when available, urine; samples were analyzed for viral load by RT-PCR and virus isolation. Whole blood was also collected from recipient ferrets at euthanasia for measurement of serum antibody against NiV. Tissues collected at post mortem were analyzed by RT-PCR, virus isolation, histopathology, and immunohistochemistry (IHC) using routine methods [13, 16]. For the time-course study, respiratory tissue sampling was more extensive. This included: upper respiratory tract (URT) comprising extra- and intrathoracic sections of the trachea, hard and soft palate (oral and nasal mucosal surfaces), nasal turbinate, pharynx, larynx, tonsil, and the base of the tongue (for pharyngeal mucosa); and lower respiratory tract (LRT) comprising hilar and peripheral regions of each major lung lobe plus hilar samples from intermediate lung lobes. All respiratory tissues were assessed by histopathology and IHC. Lower respiratory tract tissues, pharynx, nasal turbinates and trachea were also assessed by RT-PCR.

Extraction of viral RNA from nasal washes, oral and rectal swabs, whole blood and urine was carried out as described [13], or using a magnetic bead-based robotic liquid handling platform (Janus Integrator Platform, PerkinElmer, MA, USA) and MagMAX Express-96 Magnetic Particle Processor as per the manufacturer instructions (Applied Biosystems, CA, USA). Evaluation of RNA samples by multiplex RT-PCR, using primers targeting the NiV N gene and host 18S ribosomal RNA (rRNA), was performed as described [12]. Samples were considered positive for NiV N gene by RT-PCR if the mean cycle threshold value of duplicate reactions was <38.14. Virus isolation by titration on Vero cells was attempted on clinical and tissue samples positive for NiV by RT-PCR [10].

Sera from recipient ferrets were tested for the presence of a specific (binding) antibody response against NiV glycoprotein G by multiplex bead-based Luminex array (Bio-Plex, Bio-Rad Laboratories, CA, USA), and for the presence of NiV-neutralizing antibodies by serum neutralization testing (SNT; [17]). Selected sera were also assessed for differential IgM and IgG antibody responses to NiV-BD infection. To this end, binding antibody Luminex analysis was performed using methodology described by Bossart et al. (2007), except that instead of protein A/G, each sample was tested using biotinylated anti-ferret IgM and anti-ferret IgG (1:50,000 and 1:10,000 in PBS, respectively; Rockland Immunochemicals, PA, USA).

### Case definitions

Ferrets were considered to have become infected if viral RNA was detected in any tissue sample by RT-PCR at post mortem, or if anti-viral antibody was found in serum. Because analysis of tissues by RT-PCR did not allow differentiation between viral genome and mRNA representing viral transcription, viral replication was considered to have occurred within a tissue if both host and viral elements were seen: viral RNA was recovered and NiV antigen was identified within cells; or, virus was re-isolated and antigen was identified within cells; or, virus was re-isolated and histopathologic lesions typical for NiV infection (for example vasculitis, necrosis, or syncytia) were identified; or, antigen was identified in association with lesions typical for NiV infection. Respiratory secretions were defined as nasal wash and oral swab samples; viral shedding in respiratory secretions was defined as agent detection by both RT-PCR and isolation from either sample. Fever was defined as a rectal or subcutaneous temperature >40°C.

### Statistical analysis of data

For samples assessed by RT-PCR, data were expressed as NiV N gene copy numbers relative to 18S rRNA copies, which were calculated as previously described [12]. Statistical analyses were carried out using IBM SPSS statistical software (version 21.0, IBM, Foster City, CA, USA), to a significance level P ≤ 0.05 for all tests.

#### Time-course study

Residual maximum likelihood (REML) linear mixed model analyses were carried out on log transformed data of viral genome levels in respiratory secretions (nasal wash and oral swab samples), upper respiratory tract (pharynx, nasal turbinates and trachea), lungs, lymphoid tissue (spleen and retropharyngeal, submandibular and bronchial lymph nodes), and major organs (liver, kidney, adrenal gland and brain). Separate models were run for each of these tissue groups, within which, the mean NiV N gene copy number calculated from each tissue sample from each individual ferret constituted a single data point. Terms fitted to the models as fixed effects were ‘virus’ (infection group NiV-BD or NiV-MY); ‘day’ (day of euthanasia for each animal); ‘sample’ (tissue type or, in the case of lungs, specific area of each lobe sampled); and ‘fever’ (presence or absence of fever at euthanasia). The term ‘animal’ (individual ferret number) was fitted as a random effect. The interaction between ‘virus’ and ‘day’ was included for comparison of NiV-MY and NiV-BD viral loads in samples over time. The interaction between ‘virus’ and ‘sample’ was also incorporated to assess whether levels of viral RNA in different tissues varied between the two viruses. Interactions that were not significant were removed from the models prior to final analyses. For analysis of viral RNA levels in the lungs, an additional REML model was run by fitting ‘virus’, ‘day’, ‘lung region’ and ‘fever’ as fixed effects, and ‘animal’ as a random effect. This model grouped lung samples as either hilar (those areas of the lungs closely associated with the larger airways) or peripheral (those regions of the lung lobes associated with terminal bronchioles and alveolar structures).

For analysis of viral RNA levels in blood, univariate analysis of variance (ANOVA) was carried out on log transformed data using the variables ‘day’, ‘virus’ and ‘fever’ as defined for REML models above.

Contingency analyses (Fisher’s exact test) were used to assess the significance of differences between the proportion of detection of shedding of the two virus strains in respiratory tract secretions, and replication of the two virus strains in URT and LRT tissues, during both incubation (ferrets that were afebrile at the point of euthanasia) and clinical disease (ferrets with fever that was accompanied by other clinical signs in some cases).

#### Transmission studies

Contingency analyses (Fisher’s exact test) were used to assess the significance of differences between the proportion of transmission events under the two exposure conditions and with the two virus strains.

## Results

### Time-course study

All ferrets were successfully infected with either NiV-BD or NiV-MY, with one animal exposed to NiV-BD (Ferret 11) reaching its humane endpoint on d6pi, prior to its scheduled euthanasia of d7pi. Clinical disease was not observed in ferrets euthanized on days 1 to 3pi. Fever was recorded from d4pi in 6/8 remaining ferrets given NiV-BD and from d5pi in 6/6 remaining ferrets given NiV-MY. Other clinical signs included reduced play activity in NiV-MY ferrets from d5pi and agitation, disorientation, ataxia, facial edema, hunched posture, tachypnea/ dyspnea and straining to defecate in NiV-BD ferrets from d6pi.

Virus replication and shedding data are summarized in Tables 1 and 2.

**Table 1:** NiV-BD time-course study: viral shedding in respiratory tract secretions, viral genome in blood, and virus replication in tissues. For Tables 1 and 2. Lymph nodes are the retropharyngeal, submandibular and bronchial nodes. Other organs are liver, kidney, testis, and adrenal gland. ‘●’ denotes a positive result. Dpi, days post infection; RT sec, respiratory tract secretions; URT, upper respiratory tract; LRT, lower respiratory tract.

Dpi	1		2		3		4		5		6			7
Ferret No.	4	9	1	10	6	7	3#	12	5	8#	2#	13#	11#	14#
Blood^§^										●	●	●	●	●
RT sec					●	●	●	●	●	●	●	●	●	●
Rectal swab														
Urine													●	
URT					●		●	●		●	●	●	●	●
LRT	●	●	●	●	●		●	●	●	●	●	●	●	●
Lymph nodes					●	●	●	●	●	●	●	●	●	●
Spleen								●	●	●	●	●	●	●
Other organs									●	●	●	●	●	●
Brain													●	●

§Viral RNA (by RT-PCR analysis) in whole blood samples

# Fever was detected at euthanasia

**Table 2:** NiV-MY time-course study: viral shedding in respiratory tract secretions, viral genome in blood, and virus replication in tissues.

Dpi	1		2		3		4		5		6		7	
Ferret No.	23	28	19	26	18	27	16	17	21#	25#	15#	20#	22#	24#
Blood^§^										●	●	●	●	●
RT sec							●		●	●				
Rectal swab														
Urine					NS	NS				NS				
URT									●	●	●	●		●
LRT					●		●	●	●	●	●	●	●	●
Lymph nodes					●	●	●	●	●	●	●		●	●
Spleen									●	●	●	●	●	●
Other organs									●	●	●	●	●	●
Brain														

§ Viral RNA (by RT-PCR analysis) in whole blood samples

# Fever detected at euthanasia

NS, no urine sample obtained

Virus replication was detected in the LRT of NiV-BD animals from d1pi, but not in NiV-MY ferrets until d3pi. From d3pi, NiV-BD ferrets shed virus in respiratory tract secretions and replicated virus in the URT, and both groups replicated virus in lymph nodes. NiV-MY was identified in respiratory tract secretions (but not URT tissues) on d4pi and 5pi but not thereafter, in spite of ongoing URT replication in the same animals. Overall, there was a trend towards earlier recovery of NiV-BD from respiratory secretions, URT, and LRT compared to NiV-MY (P = 0.051). Among ferrets that were febrile at euthanasia, the proportion of animals shedding virus in respiratory tract secretions was significantly higher for those exposed to NiV-BD, compared to NiV-MY (P = 0.030), despite similar rates of detection of replication of the two virus strains in the URT and LRT tissues. For both strains, detection of viral RNA in blood coincided with replication of virus in spleen from d4 to d5, followed by widespread viral replication in liver, kidney, and/or gonads. In this study, virus replication in brain was only found in ferrets infected with NiV-BD.

**Fig 1:**
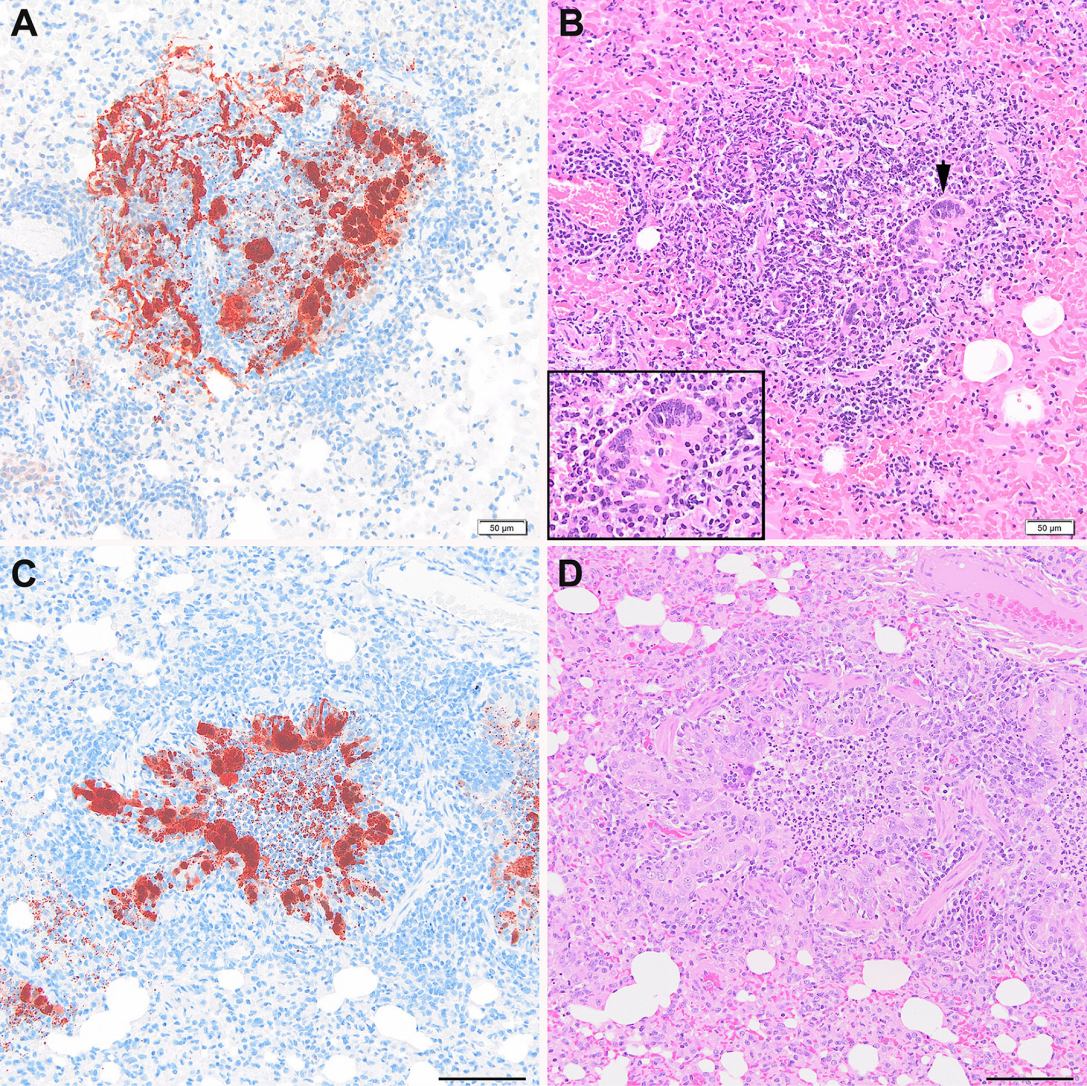
Pulmonary lesions after exposure to NiV-BD or NiV-MY. Acute bronchiolitis with epithelial syncytia and viral antigen within cells and luminal debris in a ferret exposed to NiV-MY, d3pi (A, B) and a ferret exposed to NiV-BD, d4pi (C, D). An enlarged image of the epithelial syncytium marked with a black arrow-head in panel B is also shown (inset). Once developed, features of lung pathology were comparable between the virus strains. Images (A) and (C) are IHC stained using polyclonal anti- NiV N protein; (B) and (D) are respective serial sections stained by H&E. All images 20 x original objective; scale bars for images (C) and (D), 100 um.

Once developed, the features of LRT pathology were comparable between NiV-BD and NiV-MY. Early changes were confined to accumulation of viral antigen in alveolar walls and vascular endothelium without other lesions, progressing to focal or multifocal bronchoalveolitis (Fig 1) followed by necrosis and vasculitis. Viral antigen was detected in bronchoalveolar and glandular epithelium and luminal airway debris, as well as within alveolar walls, endothelial cells, and epithelial and endothelial syncytia.

**Fig 2:**
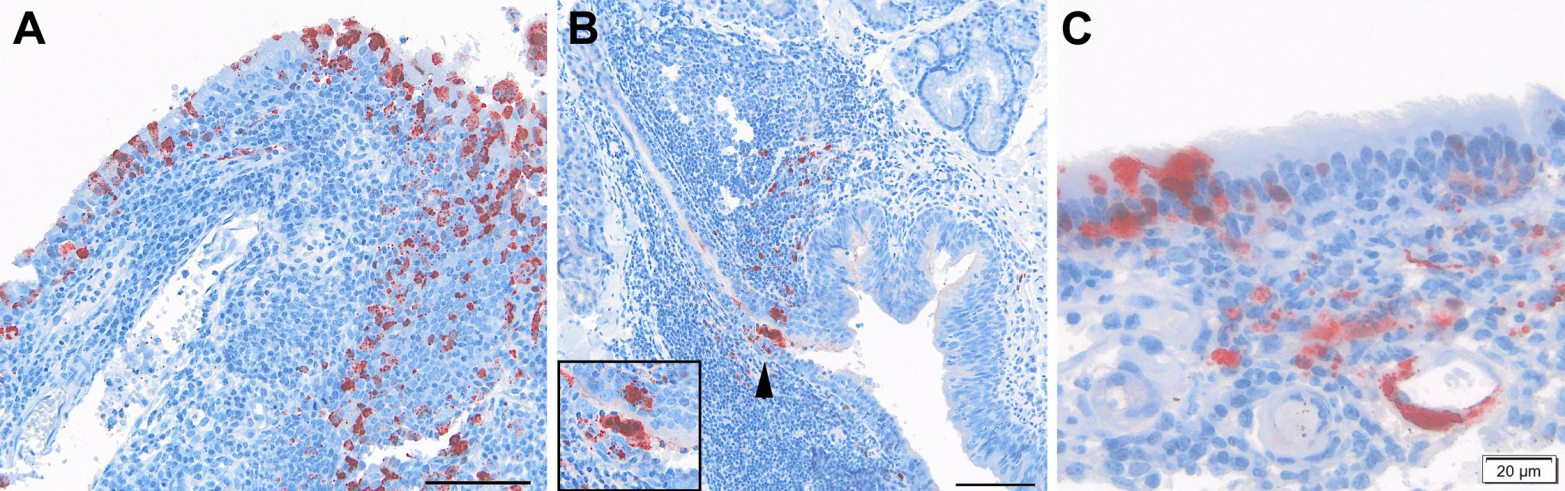
Intranasal lesions after exposure to NiV-BD or NiV-MY. (A) Viral antigen in inflamed nasopharyngeal epithelium and submucosal cells of a NiV-BD-infected ferret, d5pi. (B) Rare immunopositive staining in epithelium (arrow-head; enlarged in inset) and underlying lymphoid tissue in the pharynx of a NiV-MY-infected ferret, d5pi. (C) Viral antigen in the respiratory epithelium, submucosal cells and endothelium of the nasal cavity of a ferret exposed to NiV-BD, d7pi. (A), (B): 20x objective, scale bars 50 um; (C): 40x objective.

Similarly for the URT, acute viral rhinitis and/or nasopharyngitis were manifest on d3pi in the NiV-BD group and from d5pi in the NiV-MY group (Fig 2). Epithelial cells positive for viral antigen were also seen in the hard and/or soft palates and trachea of animals infected with NiV-BD (Ferrets 2, 8, 11, 13) and NiV-MY (Ferret 24) late in the disease course. Interestingly, at the later time points there was also viral antigen in, variously, nasopharyngeal epithelium; nasopharyngeal submucosa including connective tissues (Ferret 21) and vascular endothelium; mucosal lymphoid tissue; and tracheal and soft palate stroma of animals infected with NiV-BD (Ferrets 8, 11, 13, 14) and NiV-MY (Ferrets 15, 20, 21, 24, 25).

**Fig 3:**
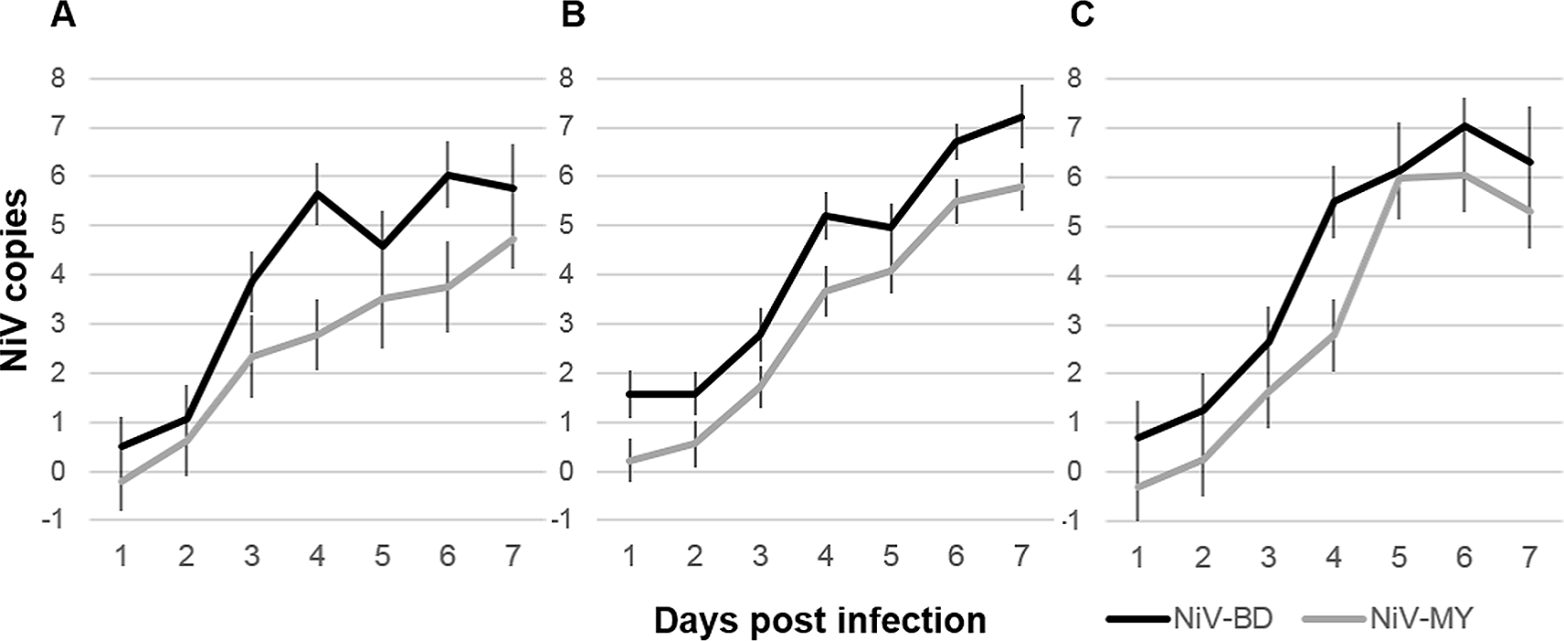
Viral loads over time in airways, lungs, and lymphoid tissues from NiV-infected ferrets. Predicted mean viral RNA levels (NiV N gene copies) over time by linear mixed model analysis of (A) airways (nasal turbinates, pharynx, proximal trachea, distal trachea); (B) lungs; and (C) lymphoid tissues (spleen and retropharyngeal, submandibular and bronchial lymph nodes) over time. Viral RNA levels increased over time, a trend that was similar between the viruses. Overall, significantly higher levels of viral RNA were recovered from NiV-BD-infected animals. Nipah virus N gene copies are presented as log_10_ copies per 10^9^ copies host 18S ribosomal RNA. Analyses were performed on log-transformed data; results are presented relative to the original scale. Error bars represent ±2 standard errors of the mean (SEM).

Viral loads in URT and LRT tissues, calculated from RT-PCR data, are presented in S1 Fig; outcomes of REML analyses of respiratory and lymphoid tissue and respiratory secretions are presented in S1 Table. Ferrets exposed to NiV-BD had significantly higher predicted mean viral RNA levels in airways, lungs and lymphoid tissues over time compared to those exposed to NiV-MY (Fig 3). For both groups, there was also a strong trend of increasing viral RNA levels in these samples as dpi progressed. Ferrets that had fever at euthanasia had significantly higher viral loads in lymphoid tissues for both viruses, a finding that was not observed for other tissues once the strong effect of day was accounted for in the statistical models (S1 Table). This finding was of unclear pathogenic significance but may reflect the extent of macrophage/ lymphocyte activation and production of pyrogenic cytokines with increasing viral loads.

**Fig 4:**
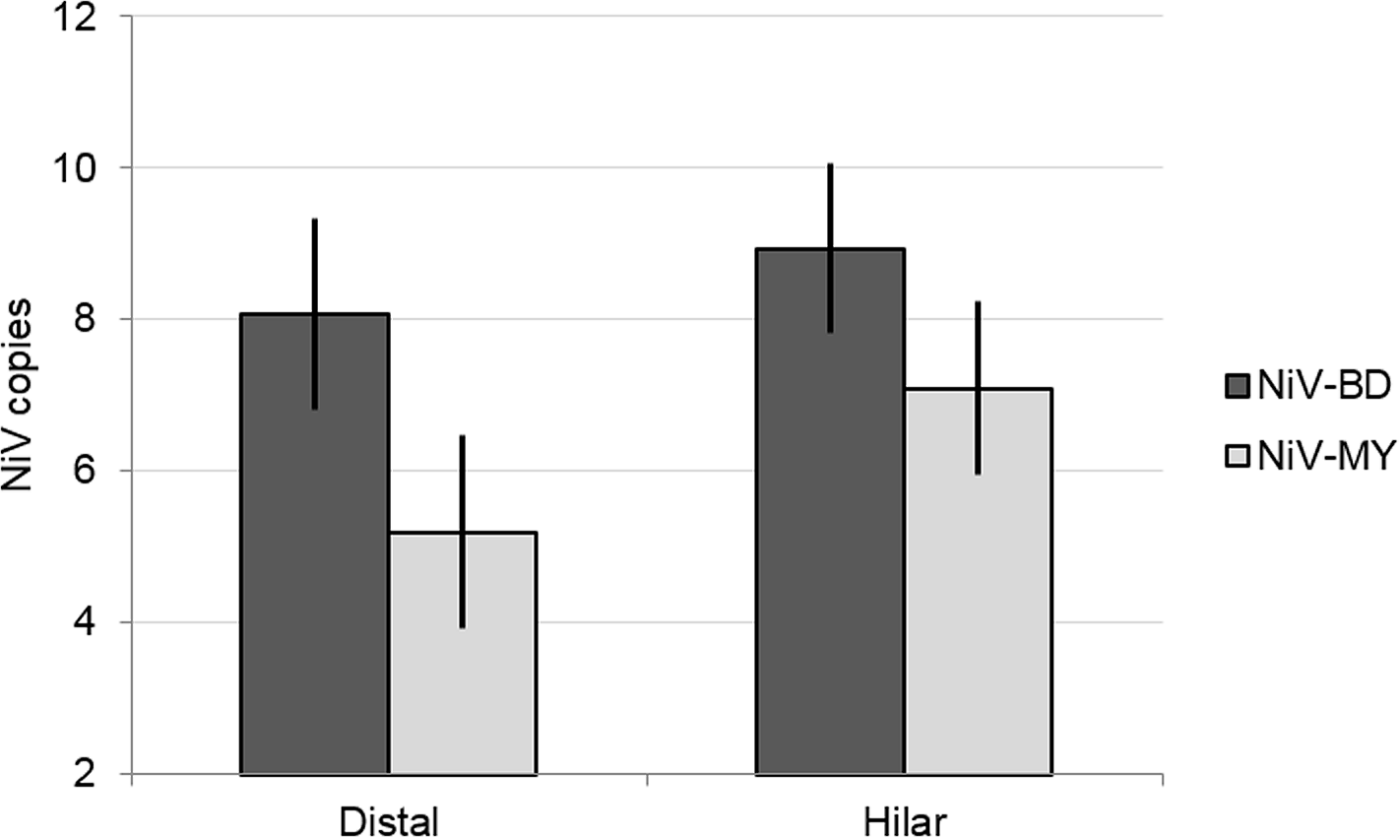
Viral load in hilar and peripheral lung samples from NiV-infected ferrets. Results of linear mixed model analysis of viral RNA levels (NiV N gene copies) recovered from different sampling sites in the lungs, when lung samples were grouped according to proximity to the hilus of the lung. In addition to the significant effect of virus, higher mean viral loads were predicted for samples collected from regions of the lung that were in close proximity to major airways (hilar), compared to those from the lung periphery (distal), for both viral strains. Nipah virus N gene copies are presented as log_10_ copies per 10^9^ copies host 18S ribosomal RNA. Error bars: ±2 SEM.

In the lungs, for both viral strains significantly higher levels of viral RNA were recovered from hilar lung tissue, compared to peripheral lung tissue samples (Fig 4).

**Fig 5:**
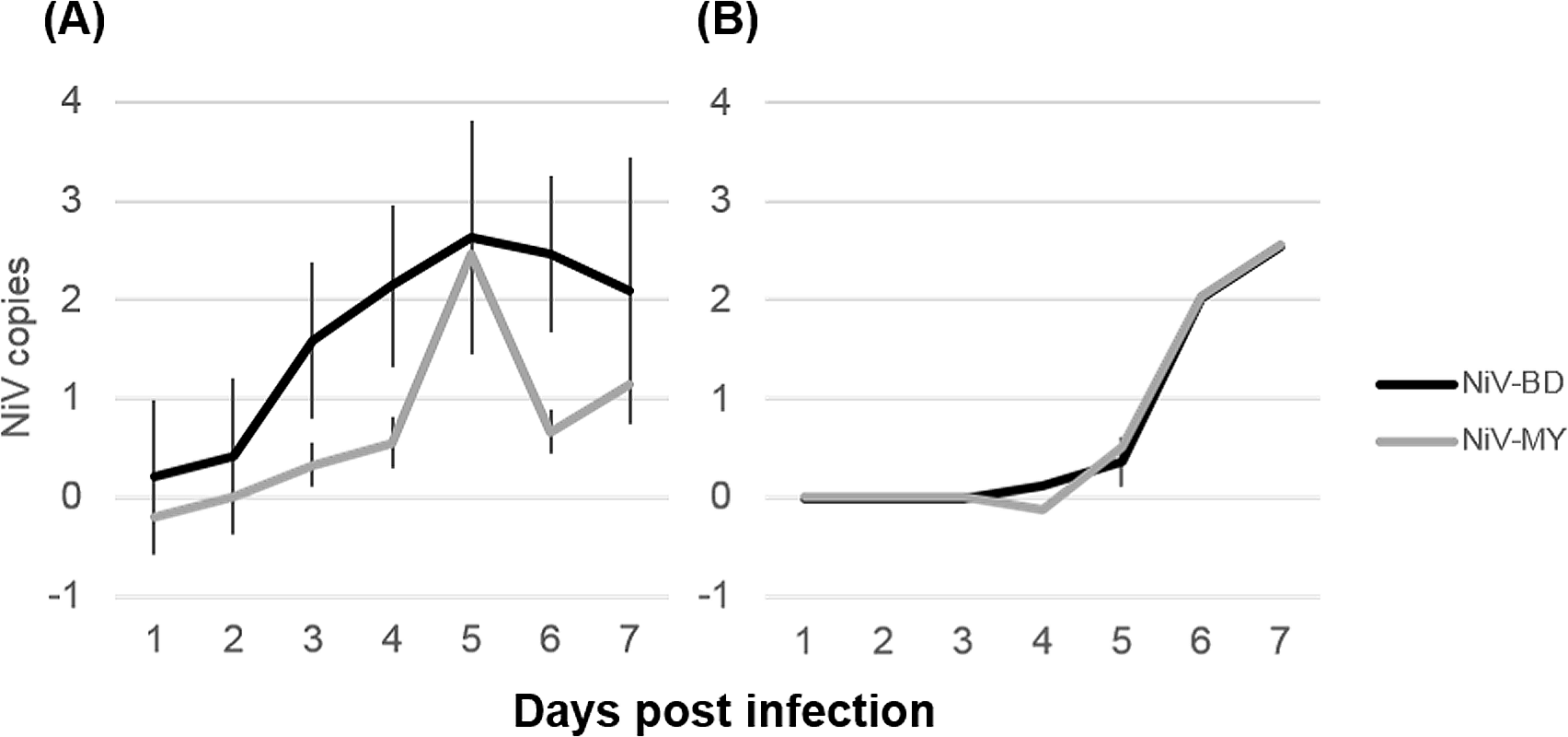
Viral loads in clinical samples from NiV-infected ferrets. Predicted mean viral RNA levels (NiV N gene copies) over time in respiratory secretions (A) by linear mixed model analysis and blood (B) by univariate ANOVA. Significantly higher levels of viral RNA were recovered from respiratory secretions of NiV-BD-infected ferrets. Levels in blood increased significantly over time but remained comparable between the infection groups (effect of virus F_degrees freedom_ = 0.001_1_, P = 0.97; effect of day F = 9.6_6_, P<0.001). Nipah virus N gene copies are presented as log_10_ copies per mL sample. Results are presented relative to the original scale. Error bars ±2 SEM.

Viral RNA was detected in nasal wash and oral swab samples from animals in which there was no evidence of viral replication in tissues of the URT, and was considered reflective of virus with a LRT origin; accordingly, nasal wash and oral swab samples were analyzed together. Compared to tissues, viral loads in respiratory secretions did not show the same strong effect of day on increasing viral load, with an increase observed to day 5 followed by a plateau or decrease in levels (Fig 5). However, as for respiratory tract tissues, predicted mean viral loads in respiratory secretions over time were significantly higher for NiV-BD-infected ferrets (Fig 5).

Levels of viral RNA in blood and in other major organs (kidney, adrenal gland, liver, thymus and brain) were comparable for the virus strains (blood, Fig 5; major organs, S2 Fig).

### Transmission study–cohabitation

Directly exposed donor animals were observed to interact closely with in-contact animals for the first two to three days of cohabitation. They engaged in play (wrestling and mouthing) and mutual grooming (including licking); slept together with shared bedding; and shared toileting spaces, food and water bowls, food items, and chew toys. With the progression of infection, each donor exhibited increasingly reduced play activity and reluctance to leave the bedding area.

All ferrets directly exposed to NiV developed clinical disease consistent with acute, systemic NiV infection and were euthanized at humane endpoint on d7pi and d8pi (NiV-BD) and d7pi and d8pi (NiV-MY). NiV was re-isolated from respiratory secretions and urine samples collected from each donor at euthanasia and histopathologic lesions consistent with acute, systemic NiV infection were found in each animal [10–12]. Virus replication was detected in URT and LRT tissues.

For NiV-BD, all four in-contact ferrets remained clinically well over the four week observation period. Viral RNA was not detected in clinical samples from any in-contact ferret at any sampling time after commencing cohabitation with a donor ferret. At post mortem examination, all tissues were negative for viral RNA, antigen, and histological lesions, including the olfactory pole and occipital region of the brain; serum was negative for anti-NiV antibody. It was concluded that NiV-BD infection did not occur in any in-contact ferret.

For NiV-MY, all four in-contact ferrets remained clinically well over the four week observation period. Viral RNA was detected in nasal wash samples of three of four ferrets on one of either 2, 4 or 6 days after commencing cohabitation with a donor ferret. The in-contact ferret which had viral RNA in its nasal wash at day 6 following cohabitation also had viral genome in a rectal swab sample at day 2. NiV was not re-isolated from RNA-positive specimens. All other clinical samples from in-contact ferrets were negative for viral RNA. At post mortem examination, all tissues, including the brain, were negative for viral RNA, antigen, and histological lesions, and serum was negative for anti-NiV antibody by Luminex assay and by SNT. Opportunity for contact with donor ferrets continued for at least 4 to 5 days (depending on the time of donor euthanasia) after cohabitation commenced. Accordingly, viral RNA in day 2 and day 4 nasal wash samples were attributed to sub-infectious viral doses or non-viable viral genome derived from donor secretions. It is possible that the in-contact ferret with viral RNA in a rectal swab at day 2 and nasal wash at day 6 (one day after donor euthanasia) experienced transient URT infection that was rapidly cleared by innate immune mechanisms. However, in the context of the current work, it was concluded that NiV-MY infection did not occur in any in-contact ferret.

### Transmission study–cohabitation plus direct transfer of secretions

Directly exposed donor animals were observed to interact closely with in-contact animals for the first day of cohabitation. With the progression of its infection, each donor exhibited increasingly reduced play activity and reluctance to leave the bedding area over the following one to three days.

All donor ferrets directly exposed to NiV developed clinical disease consistent with acute, systemic NiV infection and were euthanized at humane endpoint on either d7pi (NiV-BD) or d8pi and d9pi (NiV-MY). NiV was re-isolated from the respiratory secretions and urine samples collected from each donor at euthanasia and histopathologic lesions consistent with acute, systemic NiV infection were found in each animal [10–12]. Virus replication was detected in URT and LRT tissues.

**Fig 6:**
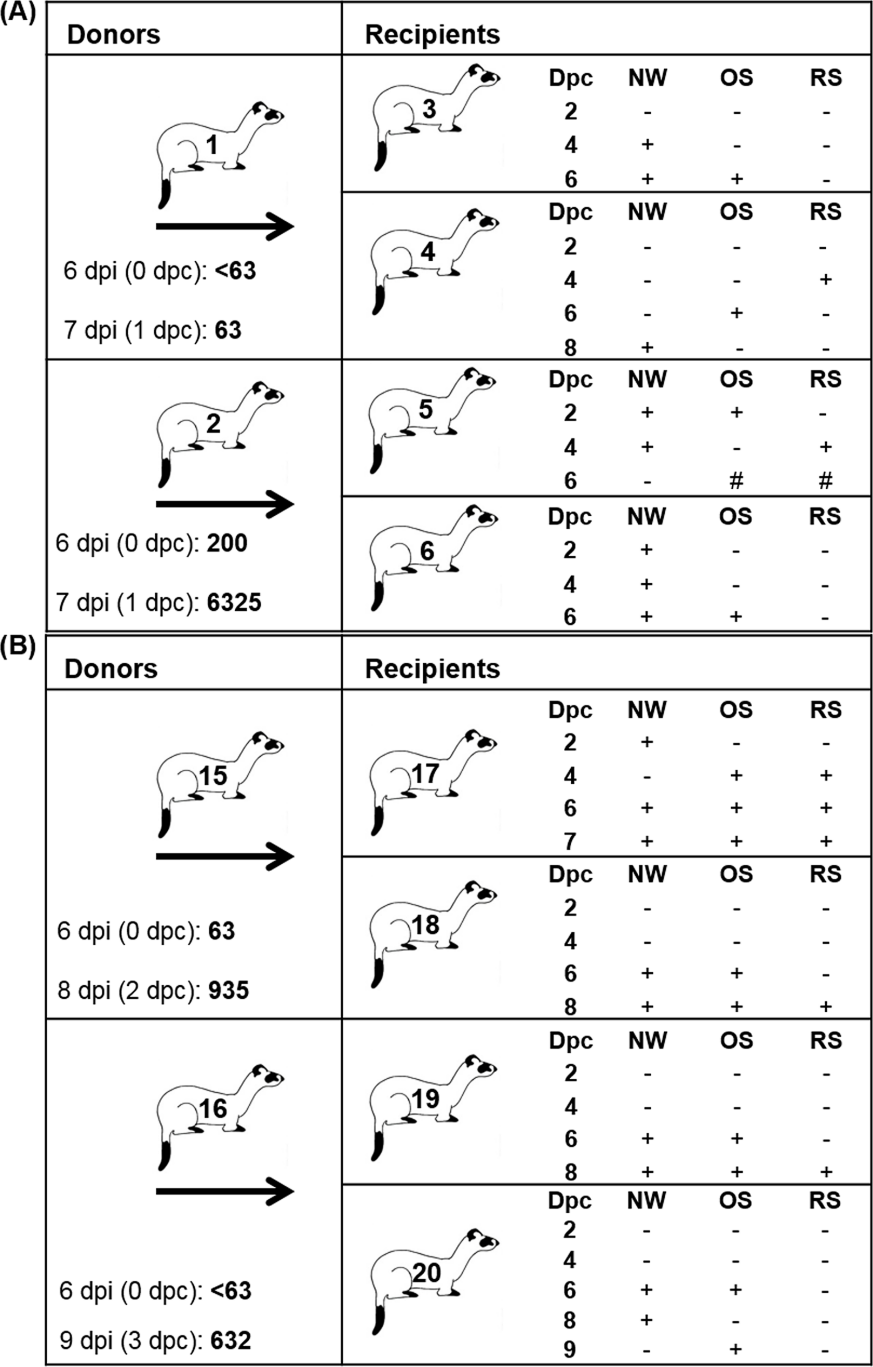
Transmission study–cohabitation plus direct transfer of secretions: Infection outcomes. Animals are presented by cage groupings for the NiV-BD (A) and NiV-MY (B) infection groups. Donors were each housed with two recipients (arrows). Recipients received their first direct exposure dose at 6 days following infection of donors and a second dose at the time of euthanasia of donor ferrets; the infectious dose (total 50% tissue culture infective dose) for each exposure event is given with the day post infection (dpi) on which the donor was sampled, and, in parentheses, the corresponding day post commencement (dpc) of cohousing of recipients. Viral shedding by recipients was assessed over the course of the study; + denotes a positive sample. Dpi, days post-donor infection; NW, nasal wash; OS, oral swab; RS, rectal swab; #, sample positive by RT-PCR but virus isolation not attempted.

One donor ferret in the NiV-BD group was febrile when recipient animals were introduced at d6pi; the other three donors (one NiV-BD and both NiV-MY donors) were not. The two virus exposure doses for each recipient pair are presented in Fig 6; titres were comparable for the virus strains and, as expected, viral titre in secretions collected at the time of donor euthanasia were equivalent to or higher than those from samples earlier in the donor infection course.

All in-contact ferrets developed clinical disease consistent with acute, systemic NiV infection reported after routine experimental exposure; they were euthanized at humane endpoint on days 6 to 9 after first exposure to donor-derived inoculum and the commencement of cohabitation. All in-contact ferrets shed virus in nasal washes and oral swabs and, variously, in rectal swab samples (Fig 6): the shedding pattern was comparable to those observed in ferrets receiving routine experimental exposure to NiV [12]. In one NiV-MY in-contact ferret (Ferret 17), respiratory shedding was detected as early as day 2 after the first exposure and prior to the second exposure to donor inoculum: infection of this animal was therefore known to have been initiated by the first donor inoculum which had a virus titer of 63 TCID_50_; the donor ferret was asymptomatic at that time.

**Fig 7:**
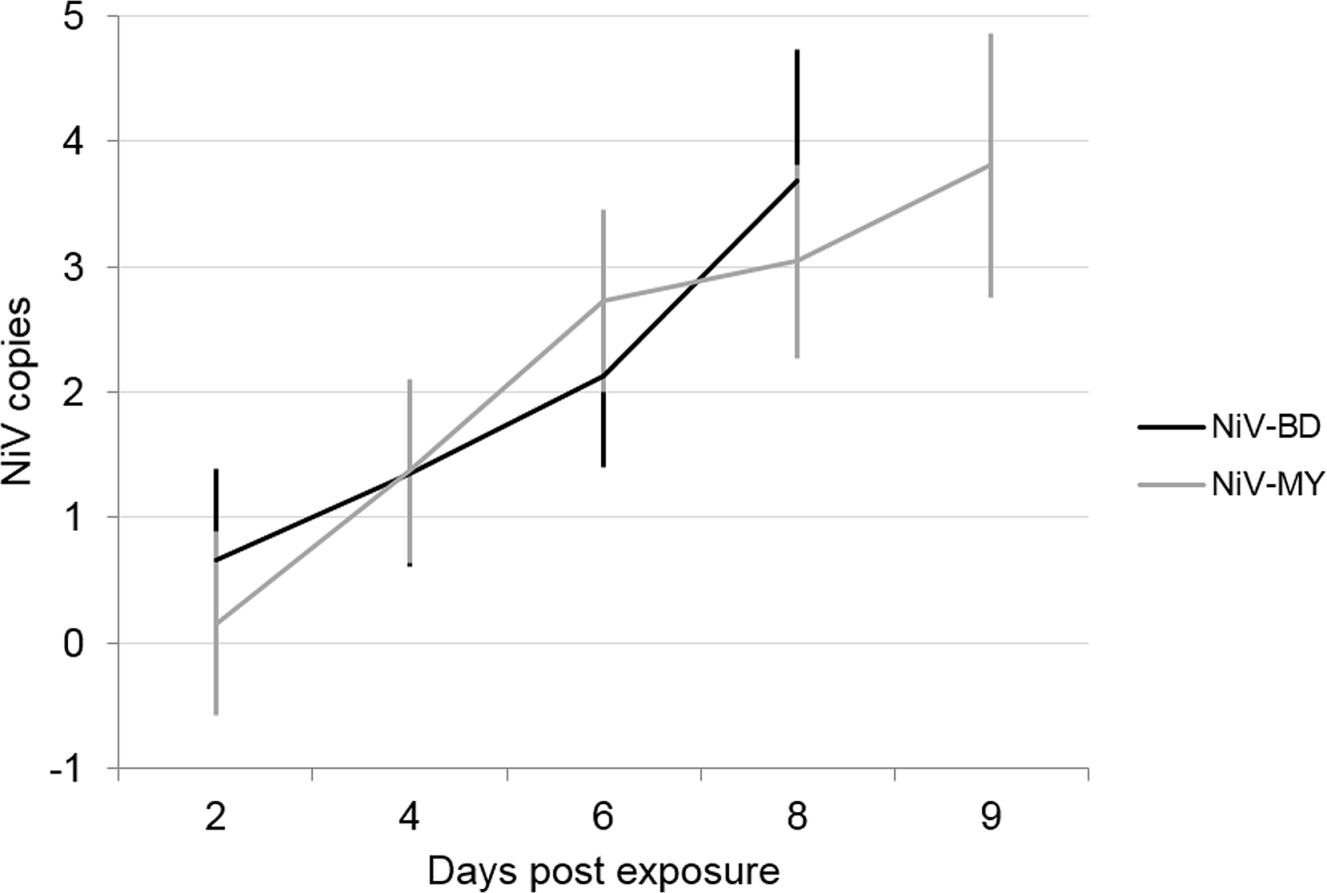
Transmission study–cohabitation plus direct transfer of secretions: Viral RNA in respiratory secretions of in-contact ferrets. Predicted mean viral RNA levels (NiV N gene copies) by linear mixed model analysis of respiratory secretions over time. Nipah virus N gene copies are presented as log_10_ copies per mL sample; predicted means are presented relative to the original scale. Error bars: ±1 SEM.

Levels of viral RNA in respiratory secretions increased across time for both viruses (Fig 7) and on this occasion were found to be comparable between NiV-BD and NiV-MY; similar viral loads were also found in lung tissues of NiV-BD and NiV-MY infected ferrets (S1 Table).

**Fig 8:**
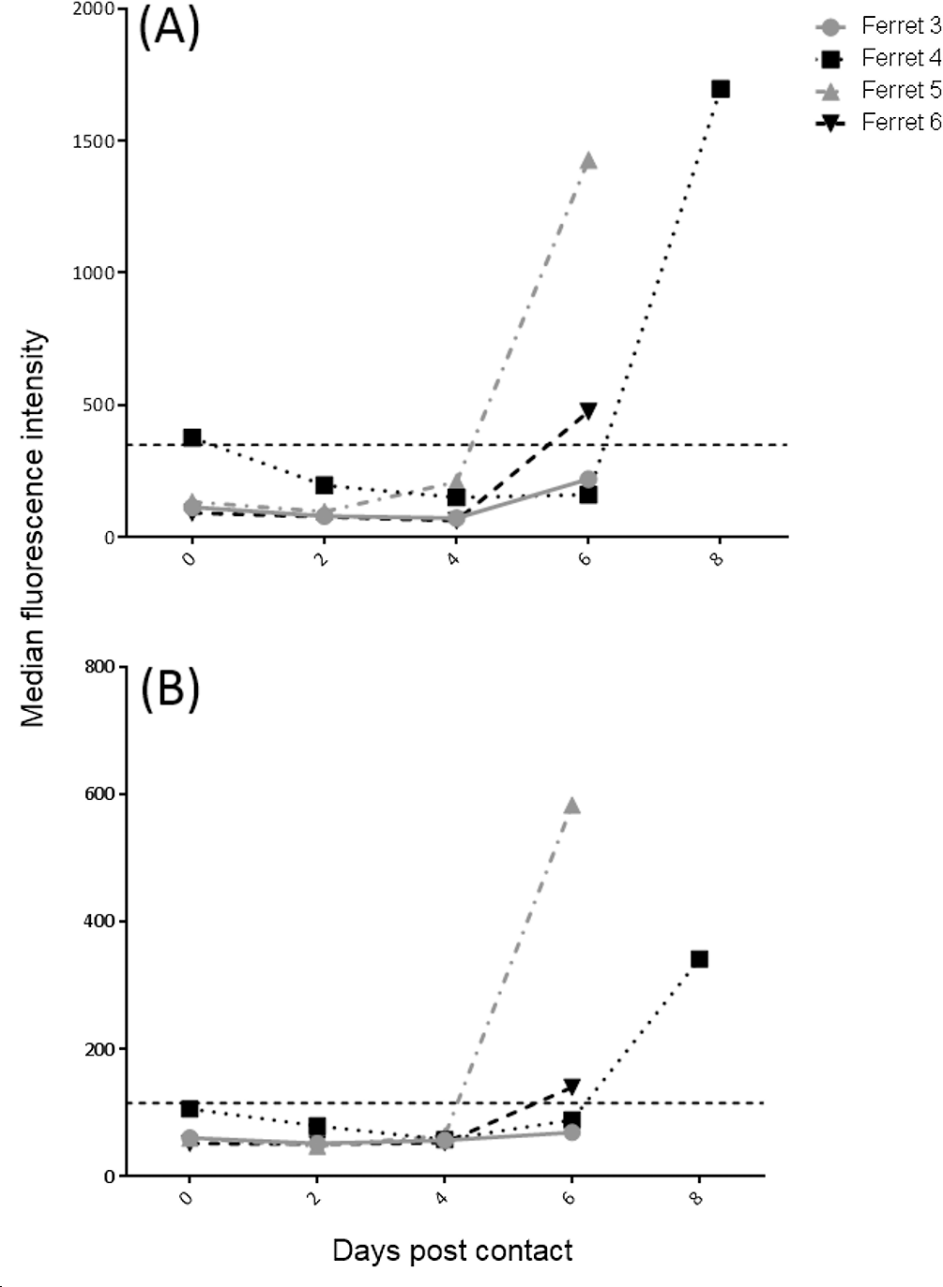
Transmission study–cohabitation plus direct transfer of secretions: Serology. Sera were tested for anti-NiV IgM (A) and IgG (B) binding antibodies on a Luminex platform. The cut-off for a positive result, represented on each of the graphs as a dashed line, was calculated for each analyte based on binding antibody results from pre-challenge serum samples collected from 17 animals in the study, which were run on the same plate with target samples to generate base-line parameters upon which cutoffs were determined. These were defined as the mean median fluorescence intensity for the 17 pre-challenge samples + 3 standard deviations of the mean. Ig, immunoglobulin.

At ferret euthanasia, all donor and recipient serum samples were negative for neutralizing antibody by SNT. Sera from NiV-BD in-contact ferrets were also analyzed by Luminex using biotinylated IgG and IgM anti-ferret antibodies (Fig 8). Three of the four animals had developed an anti-NiV IgM response between day 4 and day 8 after the first exposure to donor inoculum, accompanied by a less marked rise in IgG.

### Contingency analysis of transmission studies

There were no significant differences between NiV-BD and NiV-MY in the overall proportion of transmission events but, for each virus, the proportion of transmission events was significantly higher when direct transfer of oronasal fluids took place in addition to cohousing from late in the incubation period (P = 0.029).

## Discussion

In spite of clinical similarities in disease–but important differences in the incidence of person-to-person spread–between NiV-BD and NiV-MY, there are few reports of strain comparison studies in laboratory animals that have employed plausible natural routes of virus exposure, and reported transmission studies have been limited to NiV-MY [16, 18]. In one comparative study in which hamsters received intranasal exposure to virus, more severe lesions were identified with NiV-BD at d2pi in both URT and LRT but, by d4pi, similar degrees of rhinitis and broncho-interstitial pneumonia were present with both virus strains [19]; comparative analysis of viral loads was not described. In mice, those receiving intranasal NiV-BD were more likely to have viral antigen detected in the lungs compared those given NiV-MY [20]. However, NiV infection of mice is also subclinical and so its application to NiV strain comparisons in the context of human disease is limited, because a typically infected mouse does not exhibit equivalent illness behaviors that may influence its infectivity for other mice.

We employed an oronasal route of exposure for direct ferret challenge in this NiV strain comparison study, and it is likely that a modicum of virus inoculum was delivered directly to the ferret LRT. However, the URT was also exposed to virus at this time, providing equivalent opportunity for early replication at that site. For both NiV-BD and NiV-MY, the trend for earlier detection of infection in LRT tissues, as well as the recovery of significantly higher levels of viral RNA in hilar lung tissue suggest that under these conditions of exposure, infection is first established at the level of the bronchi or larger bronchioles. Thus, while viral replication in the URT may contribute to increasingly infectious respiratory secretions as the disease progresses, it is possible that LRT exudates may play an important role in infectivity particularly during the early stages of productive infection. Viral antigen has been noted in the bronchiolar epithelium of a human NiV case, and *in vitro* studies have demonstrated that undifferentiated primary human epithelial cells derived from bronchi and small airways were permissive to NiV infection and supported replication to high titers [21, 22]. Overall, these observations align with the demonstrated epidemiological link in NiV-BD-infected patients between coughing and an increased risk of human-to-human transmission, with coughing generating particles small enough to be inhaled into the distal airways of in-contact individuals [23].

We have shown that oronasal exposure of ferrets to comparable doses of NiV from either Bangladesh or Malaysia induced similar systemic diseases, albeit in small cohort sizes. However, we also observed a trend for earlier viral replication in the URT and LRT tissues and earlier detection of viral shedding in respiratory secretions of NiV-BD compared to NiV-MY, as well as higher levels of NiV-BD RNA in respiratory tissues and secretions over the infection course. Differences have been described for geographically-distinct NiV isolates at the genome and putative protein levels, including for the NiV-encoded nonstructural proteins V, C and W [15] which, together with P, are key viral determinants of disease outcomes in ferrets [24] and hamsters [25] infected with NiV-MY, through antagonism of host interferon-mediated innate immune responses to infection [26]. The editing site on the P gene, which prompts an open reading frame shift facilitating translation of the accessory proteins, also differs between NiV-MY and NiV-BD [15]. However, the greatest heterogeneity between NiV isolates from Bangladesh and Malaysia lies in the untranslated regions (UTRs) of the P gene encoding these accessory proteins [15, 27], and it is possible that heterogeneity within this region may give rise to differential regulation of viral gene transcriptional and translational efficiency. This has been described for mutations in gene UTRs for other paramyxoviruses [28–30] and may result in differential replicative ability of NiV at sites relevant to transmission. There is also *in vitro* evidence that viral replicative kinetics and induction of innate immune factors may differ between NiV-BD and NiV-MY, in that in hamster kidney cells NiV-MY caused more rapid and severe cytopathology and replicated to higher titers over time compared to NiV-BD [31]. However, our observations in ferrets of similar viral loads between NiV-BD- and NiV-MY in blood and other major organs, in the face of contemporaneous differences in respiratory and lymphoid tissues, suggest that functionally significant differential replicative ability of NiV strains during natural infection may be peculiar to the intact respiratory and lymphoid systems. In any event, our observations of a trend for earlier onset of respiratory tract shedding, a higher rate of respiratory tract shedding during clinical disease, and a greater magnitude of viral shedding from NiV-BD infected ferrets constitute a virus-dependent contribution to the basic reproduction number (R_0_) which might result in a difference in R_0_ between the two virus strains.

However, viral factors are only one determinant of R_0_; social factors–for example, those which define the interactions between affected individuals and the other members of the population–also play an important role in the likelihood of transmission. Accordingly, we adapted studies designed to assess the transmissibility of influenza virus strains in ferrets [32–34] to the operational constraints of the BSL-4 environment and completed two small scale comparisons of NiV strain transmissibility, providing different contact exposure opportunities which were duplicated for each virus strain.

In the first cohabitation study, we simulated such features of NiV-BD human infection clusters as: being in the same room or having close physical contact with an infected patient; sleeping in the same bed as a symptomatically-infected patient; and sharing food, cutlery or crockery with an infected patient [2, 7]. But we also observed that towards the time of peak virus replication and with the onset of clinical illness, infected ferrets reduced their play activity, food and water intake, and withdrew to their sleeping quarters from which they had to be encouraged to rise. Under these conditions of contact exposure opportunity, not only was transmission to cage contacts not observed for either NiV-BD or NiV-MY, but the data also indicate that any impact on transmission likelihood which might have been conferred by enhanced shedding of NiV-BD was not able to be shown in a study of this size.

Importantly, the interactions between sick and healthy ferrets did not adequately mirror human behaviors, as ferrets that became socially withdrawn as they became more infectious were increasingly ignored by their cage-mates. Specific activities identified as risk factors for human NiV infection in Bangladesh included direct contact with respiratory secretions from a patient, receiving a cough or sneeze from a patient directly to the face, and interaction with moribund patients through activities such as force-feeding or cradling the head of a patient [2, 9]. People who died as a result of their infection were more likely to transmit NiV, and contact with patients with advanced clinical disease was associated with a high transmission risk [2, 7]. In contrast, patients with NiV-induced disease in Malaysia received their care within advanced tertiary healthcare settings and, following detection of virus in secretions from patients early in the outbreak, hospital hygiene and infection control measures were scaled up to include rigorous barrier nursing practices to protect in-contact health care workers and patients’ visitors [35]. Considered together, these factors suggest that extremely close interactions with infected patients, in particular the direct and gross exposure of in-contact subjects to infectious material from the terminally ill, may be critical drivers of transmission.

Accordingly, in the second cohabitation study we not only simulated contact opportunity during the disease prodrome and terminal illness of infected ferrets–providing overlap with the first study in which transmission of infection was not recorded–but we also ensured the repeated exposure of in-contact ferrets to late-stage infectious secretions. This was done in order to mimic close and repeated patient contact which occurs in Bangladesh during advanced disease, intensifying as illness progresses when care-givers and family members provide increased hands-on care [9]. Under these modified conditions of contact exposure opportunity, and in contrast to the outcome of the first cohabitation study, virus transmission to all in-contact ferrets was observed for both NiV-BD and NiV-MY and with a uniformly lethal outcome. In one case, there was evidence of infection in a ferret following its first exposure to secretions from a donor that was asymptomatic, suggesting that onward transmission prior to the onset of clinical disease in patients is plausible under certain exposure conditions.

In ferrets, we found that NiV-BD replicated to higher levels than NiV-MY at anatomical sites relevant to transmission, but under the exposure conditions provided by our first cohabitation study neither NiV-BD nor NiV-MY successfully transmitted from infected to in-contact animals. More highly powered studies would be required to demonstrate smaller impacts of viral factors on R_0_ that may be of significance at the population level. However, infection is a comparatively low frequency event for both NiV-BD and NiV-MY, and the relative risk of transmission of Nipah viruses from Bangladesh and Malaysia may never be able to be experimentally defined. We also recognize that reported studies of NiV-BD to date have used a human isolate that was not associated with onward transmission in the field, and the possibility that future comparisons of additional field NiV isolates from Bangladesh may reveal even more significant differences in respiratory tract replicative efficiencies cannot be discarded. Interestingly, human-to-human transmission was reported in a recent outbreak of fatal encephalitis in the Philippines that was attributed to infection with a putative henipavirus more closely related to NiV-MY than to NiV-BD [36]. However, no virus isolate was obtained from this outbreak, and agent characterization has thus far been limited to partial genome sequencing.

Findings from the studies presented herein are consistent with the view that the risk of human-to-human transmission of certain isolates of NiV is more plausibly underpinned by environmental factors in play with each outbreak event, than by inherent differences in tissue tropism or pathogenicity between NiV strains. In particular, we have demonstrated that under experimental conditions that mimic high-risk human exposure events as reported in Bangladesh, NiV-BD and NiV-MY cause similar outcomes of infection in ferrets. Our observations suggest that a critical driver of onward NiV transmission is the nature of the interaction between subjects and that its impact may be irrespective of virus strain. In particular, the social and cultural contexts in which infection events occur, and how these influence the management of the typical infected individual, are the pivotal determinants of the likelihood of onward transmission.

## Supporting Information

S1 Fig **Detection of virus by RT-PCR in upper and lower respiratory tissues of ferrets exposed to NiV-BD (A) and NiV-MY (B), by days post infection (pi).** NiV N gene copies were normalized to 18S ribosomal RNA copies, based on standard curves generated for each target. Each individual sample was analyzed by RT-PCR in duplicate wells; data presented here are mean NiV copies per sample (error bars represent SEM). Lu, lung; L, left; R, right; Ap, apical lung lobe; D, diaphragmatic lung lobe; Int, intermediate lung lobe; dis, distal (peripheral) region of lung lobe; hil, hilar region of lung lobe; nas turb, nasal turbinates; trach, trachea; prox, proximal; thor, thoracic.
(PDF)

S2 Fig Predicted mean viral RNA levels (NiV N gene copies) over time in tissues from NiV-infected ferrets, pathogenesis study. Major organs (brain, including olfactory pole; adrenal gland; liver; kidney; and thymus) were assessed by linear mixed model analysis. Results are presented relative to the original scale. Error bars ±2 SEM.
(TIF)

S1 Table Outcomes of mixed and linear modeling analyses of data from pathogenesis study^a^ and direct fluid transfer study 2^b^. (DOCX)
