# Supplementary figures and images for: The Nature of Exposure Drives Transmission of Nipah Viruses from Malaysia and Bangladesh in Ferrets
Source: PLoS Negl Trop Dis. 2016 Jun 24;10(6):e0004775. doi: 10.1371/journal.pntd.0004775 (PMC4920392; doi:10.1371/journal.pntd.0004775)

A

Day 1pi

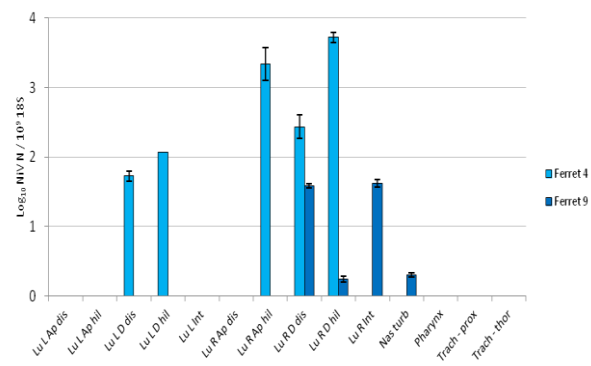

Day 2pi

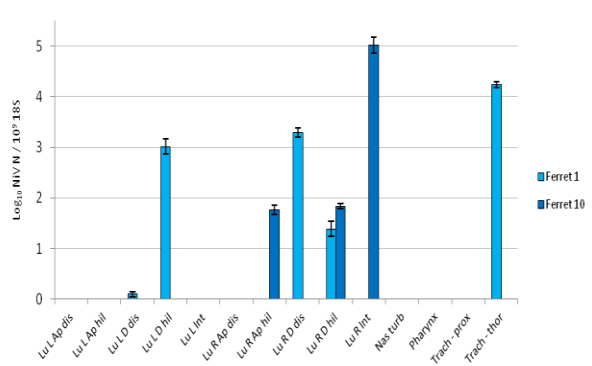

Day 3pi

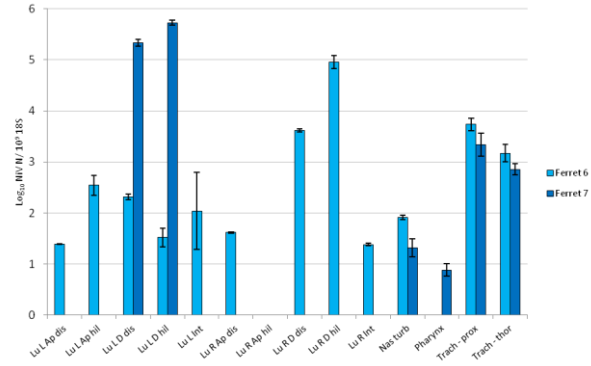

Day 4pi

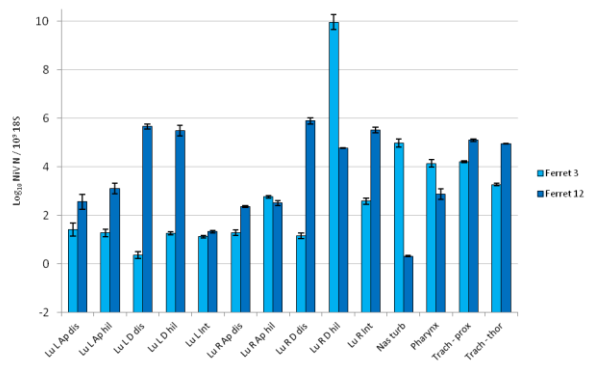

Day 5pi

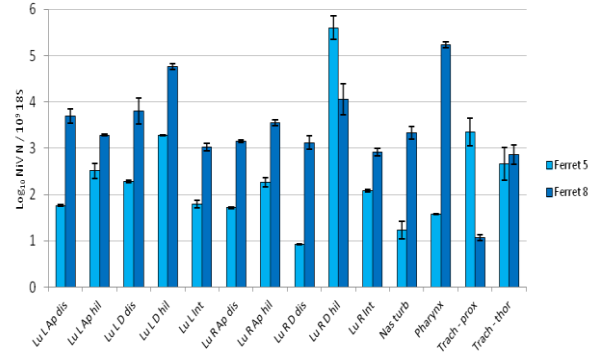

Days 6 & 7pi

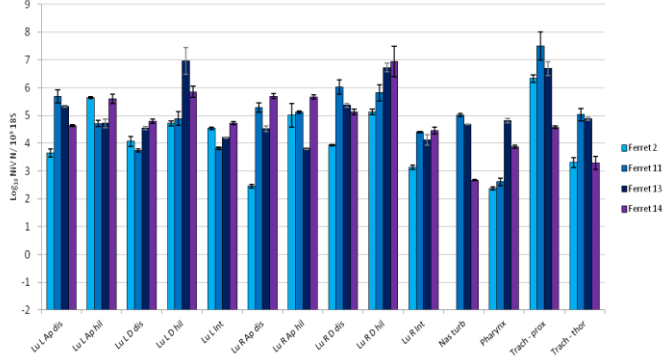

B

Day 1pi

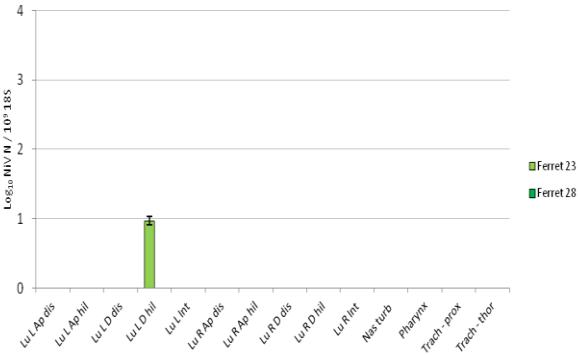

Day 2pi

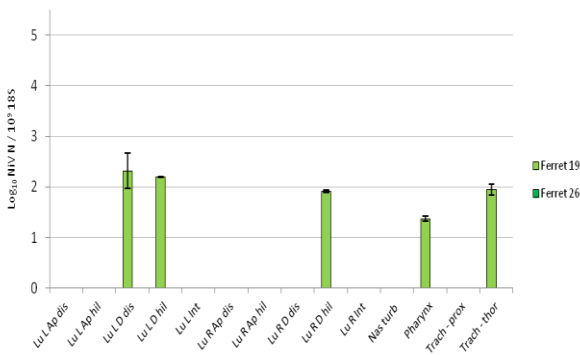

Day 3pi

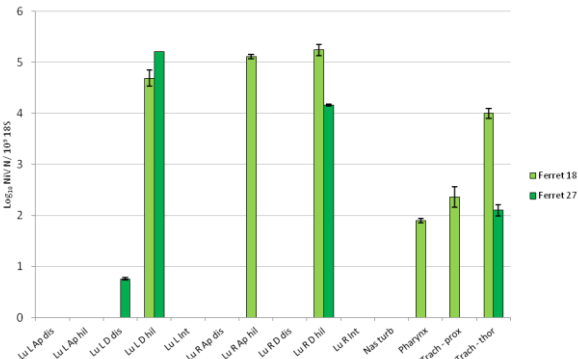

Day 4pi

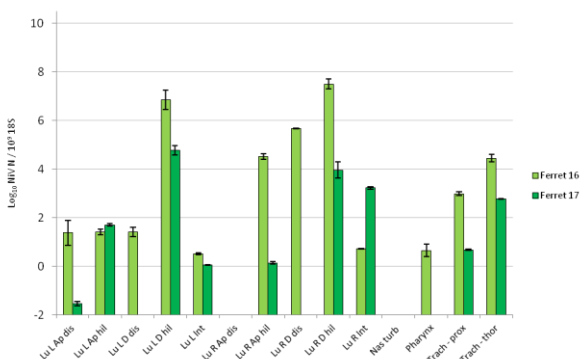

Day 5pi

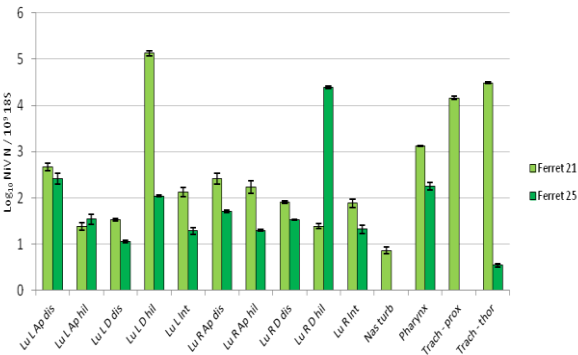

Days 6 & 7pi

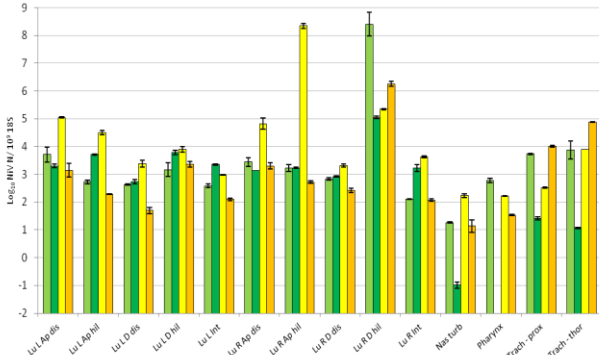

Supplement: S1 Fig — Detection of virus by RT-PCR in upper and lower respiratory tissues of ferrets exposed to NiV-BD (A) and NiV-MY (B), by days post infection (pi). NiV N gene copies were normalized to 18S ribosomal RNA copies, based on standard curves generated for each target. Each individual sample was analyzed by RT-PCR in duplicate wells; data presented here are mean NiV copies per sample (error bars represent SEM). Lu, lung; L, left; R, right; Ap, apical lung lobe; D, diaphragmatic lung lobe; Int, intermediate lung lobe; dis, distal (peripheral) region of lung lobe; hil, hilar region of lung lobe; nas turb, nasal turbinates; trach, trachea; prox, proximal; thor, thoracic. (PDF) [file pntd.0004775.s001.pdf]

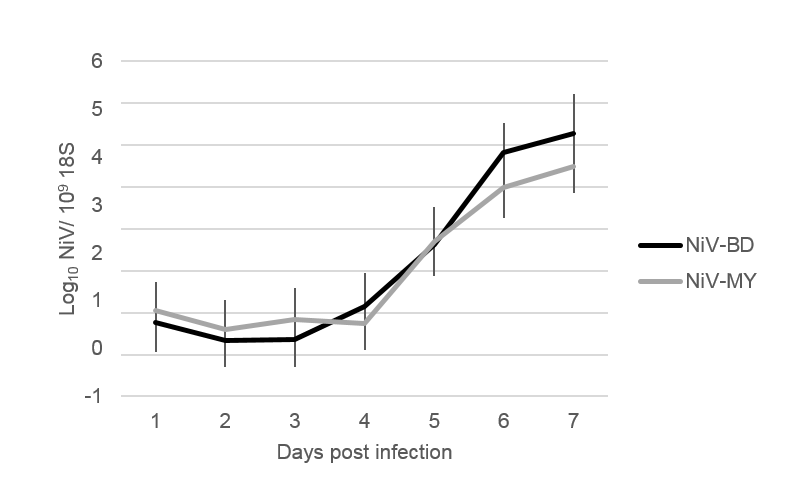

Supplement: S2 Fig — Major organs (brain, including olfactory pole; adrenal gland; liver; kidney; and thymus) were assessed by linear mixed model analysis. Results are presented relative to the original scale. Error bars ±2 SEM. (TIF) [file pntd.0004775.s002.tif]
