# Supplementary material for: The Nature of Exposure Drives Transmission of Nipah Viruses from Malaysia and Bangladesh in Ferrets
Source: PLoS Negl Trop Dis. 2016 Jun 24;10(6):e0004775. doi: 10.1371/journal.pntd.0004775 (PMC4920392; doi:10.1371/journal.pntd.0004775)
Supplement: S1 Table — (DOCX) [file pntd.0004775.s003.docx]

| **Model** | **Fixed effects** | | | **Covariances** | |
| --- | --- | --- | --- | --- | --- |
|  | Effect | F_df num/denom_ | P value | Parameter | Est (SE) |
| Lung by sample^a^ | Virus | 15.9_1/18.9_ | **0.001** | Resid | 2.6 (0.2) |
|  | Fever | 0.8_1/19_ | 0.372 | Animal | 0.3 (0.2) |
|  | Day | 8.7_6/19_ | **<0.001** |  |  |
|  | Sample | 11.2_9/244_ | **<0.001** |  |  |
| Lung by region^a^ | Virus | 16.0_1/18.9_ | **0.001** | Resid | 3.4 (0.3) |
|  | Fever | 0.8_1/19_ | 0.374 | Animal | 0.2 (0.2) |
|  | Day | 8.7_6/18.9_ | **<0.001** |  |  |
|  | Lung region | 10.6_1/252_ | **0.001** |  |  |
| URT^a^ | Sample | 9.9_3/81_ | **<0.001** | Resid | 2.4 (0.4) |
|  | Day | 3.2_6/19_ | **0.024** | Animal | 1.0 (0.5) |
|  | Virus | 7.9_1/19_ | **0.011** |  |  |
|  | Fever | 1.7_1/19_ | 0.207 |  |  |
| Lymphoid tissues^a^ | Sample | 65.5_3/96_ | **<0.001** | Resid | 4.7 (0.7) |
|  | Day | 7.8_6/96_ | **<0.001** | Animal | 0 (0) |
|  | Virus | 5.6_1/96_ | **0.020** |  |  |
|  | Fever | 4.2_1/96_ | **0.043** |  |  |
| Resp secretions^a^ | Sample | 6.1_1/26_ | **0.020** | Resid | 0.6 (0.2) |
|  | Day | 1.8_6/19_ | 0.163 | Animal | 0.4 (0.2) |
|  | Virus | 7.9_1/19_ | **0.011** |  |  |
|  | Fever | 2.8_1/19_ | 0.109 |  |  |
|  | Sample*Virus | 16.3_1/19_ | **<0.001** |  |  |
| Resp secretions^b^ | Virus | 0.3_1/2_ | 0.658 | Resid | 0.6 (0.2) |
|  | Day | 9.9_5/21.3_ | **<0.001** | Animal | 0 (0) |
|  |  |  |  | Donor | 0.3 (0.4) |
| Lung by sample^b^ | Virus | 0.7_1/3_ | 0.807 | Resid | 1.3 (0.2) |
|  | Sample | 5.0_9/63_ | <0.001 | Animal | 0.4 (0.7) |
|  | Day | 0.6_3/4.5_ | 0.641 | Donor | 0.2 (1.0) |
| URT^b^ | Virus | 0.8_1/3_ | 0.435 | Resid | 2.5 (0.8) |
|  | Sample | 1.8_3/21_ | 0.184 | Animal | 0.4 (0.8) |
|  | Day | 1.8_3/3_ | 0.327 | Donor | 0 (0) |

URT, upper respiratory tract (nasal turbinates, pharynx, trachea); resp, respiratory; df, degrees of freedom; num, numerator; denom, denominator; est, estimate; SE, standard error; resid, residual. Significant effects (P<0.05) are presented in bold type. Interactions between fixed effects (*) were included in the final models for analysis only if they were significant.
